# Risk stratification in breast screening workshop

**DOI:** 10.1186/s12919-024-00306-0

**Published:** 2024-10-24

**Authors:** Andrew Anderson, Cristina Visintin, Antonis Antoniou, Nora Pashayan, Fiona J. Gilbert, Allan Hackshaw, Rikesh Bhatt, Harry Hill, Stuart Wright, Katherine Payne, Gabriel Rogers, Bethany Shinkins, Sian Taylor-Phillips, Rosalind Given-Wilson

**Affiliations:** 1 UK NSC secretariat, Sheffield/London, UK; 2 University of Cambridge, Cambridge, UK; 3 University College London (UCL), London, UK; 4 The University of Sheffield, Sheffield, UK; 5 The University of Manchester, Manchester, UK; 6 The University of Warwick, Coventry, UK; 7 St George’s Healthcare NHS Foundation Trust, London, UK

**Keywords:** Breast cancer, Screening, Risk stratification, Epidemiology, Cost-effectiveness

## Abstract

Population screening for breast cancer (BC) is currently offered in the UK for women aged 50 to 71 with the aim of reducing mortality. There is additional screening within the national programme for women identified as having a very high risk of BC. There is growing interest in further risk stratification in breast screening, which would require a whole population risk assessment and the subsequent offer of screening tailored to the individual’s risk. Some women would be offered more intensive screening than others or no screening. This might provide a better balance of screening benefits and harms for each individual than the current population age-based programme alone. The UK National Screening Committee (UK NSC) is considering using decision-analytic and other models to evaluate different risk stratification screening strategies and identify remaining gaps in evidence. This paper reports the proceedings of a UK NSC workshop where experts in the field discussed both risk prediction models, as well as decision-analytic models providing a benefit-harm analysis/economic evaluation of risk-stratified screening programmes (see Table 1). The aim of the meeting was to present and discuss the current work of experts, including some data which had not been published at the time of the meeting, to inform the UK NSC. The workshop was not intended to present a balanced evaluation of how to deliver screening in future. Areas for further work identified included methods for comparing models to assess accuracy, the optimum risk assessment tools, the digital screening infrastructure, acceptability of stratification, choice of screening test and reducing inequalities. A move to risk stratification of the whole programme would require a careful phased introduction with continuing assessment of real-world evidence during deployment.

## Introduction

BC is the most common form of cancer in England [1]. The NHS Breast Screening Programme (NHS BSP) in England [2], consisting of regular screening with mammography, is designed to reduce BC mortality through earlier detection of cancers which might be more amenable to treatment.

Early diagnosis of BC through screening has led to BC specific mortality reduction, as well as more effective and targeted therapies [3]. However, there are populations where early detection of BC is less achievable with the current approach, such as in women with dense breasts and those of black and Asian ethnicities [4, 5]. Furthermore, studies in Europe and North America have found that there is not always a correlation between national screening coverage and a decrease in mortality [6].

There are also harms associated with screening including overdiagnosis, overtreatment and psychological morbidity. Interval cancers are inevitable and more will be seen with a 3-year screening interval as opposed to more frequent screening and in women with dense breasts [7].

Identification of women at higher or lower risk of BC (risk stratification) compared with the general population, and tailoring screening according to risk, may improve the programme’s benefits to harms ratio by focusing interventions on those who would benefit the most and reducing, or discontinuing, screening for those who stand to gain less.

The NHS BSP bases eligibility on the two most important risk factors of all – sex and age. The programme routinely invites anyone registered with a GP as female every three years between the ages of 50 years and their 71st birthday [8].

There is some risk stratification in the current NHS BSP with enhanced screening of very high risk women including those with rare pathogenic mutations such as BRCA1, BRCA2, or TP53, as well as women with a strong family history of BC leading to equivalent risk. The problem is that risk assessment is initiated by a woman presenting to a GP with a family history. Almost half of women at higher risk do not have family history and will be missed. We need population level risk assessment to identify all those who would benefit from more intensive surveillance.

Other women at moderate and high risk are managed in the UK in accordance with National Institute for Health and Care Excellence (NICE) guidance [9] albeit implementation may differ [10]. NHS England (NHSE) has a very high risk women screening surveillance protocol to guide practice [11]. There are a variety of other known BC risks which the NHS BSP does not currently use.

Introducing risk-assessment mechanisms, such as self-reporting of BC risk factors (for example family history, lifestyle/hormonal factors), assessing risk based on genetics, and/or mammographic density, on a population basis could potentially give the programme much richer information on which to stratify, opening the way for people to be invited at different ages, frequencies, and using different screening tests. However, there is still much work to do to ensure the evidence is solid for assessing risk, especially given the complexities inherent to genomic prediction of future disease risk [12]. Meanwhile, there is lack of clarity about the clinical and cost effectiveness, feasibility (for example, the requirement for staff training and IT system development) and acceptability of stratifying the NHS BSP.

## UK NSC criteria

The independent UK NSC [13] is the body responsible for making recommendations about any changes to the NHS BSP. In May 2022, it announced its expansion to consider targeted and stratified screening [14], which opened the possibility of further stratification in the NHS BSP.

There is still a lack of information to support understanding of the possible effects of a stratified NHS BSP. There have been some modelling studies, and validated BC risk prediction models such as Tyrer-Cuzick [15] and CanRisk [16].

The UK NSC requires high-quality evidence [17] to help determine whether a significant change to a screening programme is clinically effective, safe, and acceptable, and whether it represents an effective use of public funds (Table 1).

**Table 1:** Summary of primary research question and model type

Presenter	Institution leading the analysis	Date model last updated	Model type	Primary research question	Published (Y/N) and bibliography reference if any
Nora Pashayan	University College London	2021	Life-table model	In risk-targeted breast cancer (BC) screening, what are the optimal risk thresholds that could improve the benefit-harm balance and cost-effectiveness of the breast screening programme?	Y [23]
Rikesh Bhatt	University College London	2022	Multistate model and microsimulation	Which are the optimal screening risk-stratified strategies by varying starting age, ending age and frequency of screening compared with no screening or current screening?	N
Harry Hill	University of Sheffield	2024	Decision-analytic (individual-level discrete event simulation)	What is the cost-effectiveness of eight proposals for risk-stratified screening compared with both the current UK screening programme and no national screening?	Y [22]
Fiona J. Gilbert	University of Cambridge	2022	MIRAI image risk prediction model	Can information from mammogram be used to predict who will develop BC in next 5 years?	N
Antonis Antoniou	University of Cambridge	2024, CanRisk Releases	BC risk prediction model	How can we personalise BC risk based on the combined effects of established risk factors for the disease?	Y [28, 29]
Stuart Wright	The University of Manchester	2023	Decision-analytic (individual-level discrete event simulation)	Evaluation of a Risk-Stratified National Breast Screening Programme in the United Kingdom: An updated cost-effectiveness analysis. (*To note, model results were unpublished at the time of the meeting but are now available in a pre-print/pre-peer review paper)*	Y [27]

## RCTs

The gold standard evidence has traditionally come from high-quality randomised control trials (RCTs) [18] which suggest the screening programme is effective in reducing mortality, morbidity and that the benefit gained by the individual should outweigh the harms. One such trial is the cluster randomised AgeX trial [19], the largest RCT to be conducted in screening, which will assess the risks and benefits of offering an extra invitation to women below and above the current screening age. It is not expected to begin reporting mortality data before 2026.

A benefit of cancer screening RCTs – which have disease specific mortality as an end point – is that they are a better measure of important outcomes than cancer stage shift, which appears an unreliable predictor of mortality reduction [20].

RCTs typically inform cost-effectiveness analyses, by evaluating the additional costs associated with a screening programme alongside the health benefits and harms, compared with the current standard of care. However, in the context of risk-stratified screening, empirical trials can be complex, lengthy, and it is difficult, if not impossible, to assess all the possible questions relating to risk-stratified screening within a single trial. Such questions include which risk factors to stratify on, which risk assessment tools to use, which risk threshold to be selected, which risk groups to receive which tests, and how often.

Decision-analytic models can be useful to synthesise evidence from different sources (for example, test accuracy studies, model validation studies, epidemiological data on disease risk and progression). They are used even when RCT evidence is available in screening due to the often-large number of possible screening strategies, and because it will always be necessary to extrapolate beyond observed follow-up to estimate the benefits, harms, and costs with which competing approaches are associated over a cohort’s entire lifetime. Prime examples of this kind of work include the model developed for targeted screening for lung cancer, which evaluated the cost-effectiveness of 48 different screening strategies compared with no screening [21], and the change to cervical screening intervals.

Models still require data from high-quality evidence to provide reliable outcomes. They provide a useful framework though for narrowing down the number of screening strategies to those most likely to be clinically and cost-effective and identifying where the key gaps in the evidence base lie. Building a high-quality model which fully captures the complexity of risk-stratified screening is no straightforward task, and different approaches have been adopted. The UK NSC therefore needs to:

- evaluate the different approaches to modelling risk-stratified BC screening.
- understand the outputs of these models in terms of the predicted benefits, harms and costs associated with different risk-stratified screening strategies.
- identify the key gaps in the evidence and determine how to fill them.

The UK NSC organised a risk stratification modelling workshop with leading researchers in the field to consider different options available to inform future discussions.

Researchers representing some UK groups working in this area were invited to present their models and findings. They were not intended to be representative of all research within this area. All presenters had met with representatives of the UK NSC and NHSE before the session.

## Workshop presentations

### Risk-stratified screening decision-analytic models

Bhatt described a study he co-authored, unpublished at the time of the meeting, which used NHS BSP data linked to existing population-based case–control study with information on risk factors. The analysis was based on multistate survival models of the natural history of BC. Three risk categories were considered. The team simulated more than 500,000 screening strategies, varied by age range and frequency of screening for each risk category. The outcomes were compared in screening episodes, BC diagnoses, interval cancers (a BC found during the 3 years after a normal result and before the next screening appointment), overdiagnoses (the excess number of incident BC cases diagnosed over a lifetime with each screening strategy compared with no screening), life years gained, and BC mortality.

Hill presented a decision analytic economic model which evaluated the cost-effectiveness of eight proposals for risk-stratified screening regimens compared with the current UK screening programme and no screening [22]. Compared with the current screening programme, all risk-stratified regimens generated additional costs and QALYs and had a larger net monetary benefit. The findings of this study indicate risk-based screening has the potential to improve the cost-effectiveness of the NHS BSP. The model indicated that among the eight screening regimens evaluated, the most cost-effective one was the only one not offering screening to individuals with low risk. It was also estimated to be more cost-effective than the current screening program in the UK. Triennial, biennial, and annual screening among the three risk groups was the optimal screening strategy in this model.

Pashayan evaluated 99 scenarios of risk-stratified screening and found that not offering screening to women at the lowest tertile of the risk distribution would improve the cost-effectiveness, reduce overdiagnosis, while maintaining the benefits of screening [23].

Wright described a decision-analytic model built by a research team based at The University of Manchester called MANC-RISK-SCREEN to assess the cost-effectiveness of different risk-based screening programmes [24]. MANC-RISK-SCREEN is a discrete event simulation where individual women are simulated through different screening strategies. It uses the Tyrer-Cuzick questionnaire and Volpara breast density measurement to predict risk as outlined in the BC-PREDICT study [25]. Evaluated strategies include shorter screening intervals for women at high (> 8% ten-year) and moderate (> 5% ten-year) risk, and a mix of shorter screening intervals for higher risk women and less frequent screening for low-risk women (< 1.5% ten-year risk). The model builds on a published micro costing study of the cost of risk prediction [26]. MANC-RISK-SCREEN has undergone technical verification and a validation process. Wright presented model results — unpublished at the time of the meeting, but now available in a pre-print paper [27] which suggest that while more intensive screening such as 2 yearly screening or dividing women into risk tertiles are likely to be the most cost-effective strategies, the strategies based on the BC-Predict study were cost-effective compared with 3-yearly screening while requiring a similar number of mammograms.

### Risk assessment models

Antoniou presented the validated multifactorial Breast and Ovarian Analysis of Disease Incidence and Carrier Estimation Algorithm (BOADICEA) [28] model for estimating an individual’s risk of BC. This model is based on the combined effects of the known risk factors for the disease (genetic, lifestyle/hormonal, imaging risk factors, etc.) [29]. Importantly, it has been shown that the inclusion of mammographic density, improves the discriminatory and risk-stratifying ability of BOADICEA [30]. BOADICEA is implemented using the CanRisk web-based user-friendly tool [31] that has gained regulatory approval for use as a medical device. CanRisk is designed to provide personalised cancer risks which can be linked to management strategies including screening regime, and prevention options such as risk-reducing medication or surgery [32].

Gilbert described a study (unpublished at the time of the meeting) looking at the use of AI in analysing digital information from mammograms to predict which women will develop BC in the next 5 years. The Mirai model has been tested on different cohorts and showed a slight improvement compared with the traditional BC risk model of Tyrer-Cuzick. The team tested this model on an unseen retrospective dataset from Cambridge and Huntingdon breast screening programme and showed similar accuracy for predicting cancer in the next 5 years to Yala et al. [33]. Another promising model is the Karma model [34] tested on the Swedish cohort at the Karolinska. Karma is an artificial intelligence (AI) tool based on mammographic features (cancer signs, texture analysis and density) which predicts risk of developing BC within two years which has shown high discriminatory performance compared with traditional lifestyle/familial-based risk models. [35].

## Unanswered questions

Additional information is still needed before the UK NSC can make an assessment, based on its evidence review criteria, on what a risk-stratified NHS BSP might look like, and what the possible effects to the population and costs to the healthcare system might be.

One important outcome is the number of BCs that might be missed by a risk-stratified approach compared with the current screening policy, and this would be balanced against the expected reduction in the number of false-positives (women who do not have BC but are screen-positive and undergo further investigations) and in overdiagnoses.

Another important question is whether (and how) to implement routine screening in women aged 47–50 and/or 70–73 following the publication of results of the AgeX trial. Ultimately, results of the trial will provide evidence around the benefit/harm of extending the age range for routine screening. It will be possible to incorporate this information into risk stratification models which are aimed at improving mortality and reducing over diagnosis by introducing more personalised screening strategies.

### Comparing decision-analytic models

Each of the decision-analytic models differ in their structure, choice of source data, assumptions, and screening modalities, which leads to different findings. The group briefly discussed the possibility of linking the models but acknowledged that doing so would be neither be feasible nor informative.

It was suggested that the UK NSC instead evaluate the clinical and cost effectiveness of common risk-stratification approaches using the outputs of all decision-analytic models, allowing a comparison between them. This offers the potential of concordance models that the UK NSC thinks are of high quality, both in terms of assumptions and mechanisms, to strengthen the evidence base.

MANC-RISK-SCREEN was validated on external data to assess whether the model correctly predicted outcomes. Validating all the decision-analytic models on the same external datasets would provide a mechanism to compare the head-to-head performance of models and better understand which outcomes they accurately estimate, or under- or over-estimate. Validation datasets should be representative of the UK population.

### Which risk assessment tools to use

Risk-assessment models such as CanRisk and Tyrer-Cuzick have effectively used a combination of modalities to identify women at low and high risk (monogenic and polygenic risk scores, mammography density, and self-reporting). Self-reporting of BC risk factors includes lifestyle factors (alcohol), anthropometric (BMI), hormonal (HRT, OCP use), reproductive (age of menarche, age of menopause, number of pregnancies, breast feeding), and family history of BC [36].

There are unknowns about the effectiveness of some of these tools in certain populations or how they can be operationalised in practice. For example, in the case of mammographic density, there is strong and consistent evidence [37] that dense breasts increase the risk of BC and decrease the sensitivity of mammography to detect cancers.

Taking account of mammography density in the breast screening programme would require a reliable method to assess breast density with a standardised definition of high mammographic breast density, and clear evidence on the most effective screening test for women with increased density. Baseline imaging will be needed to measure mammographic density and could be incorporated into AI supported risk prediction based on imaging factors.

It is important to recognise the complexities of pathway design and flow when considering the feasibility and desirability of implementing risk-stratified screening in the NHS BSP. Studies such as Breast Screening Risk Adaptive Imaging for Density (BRAID) [38] could be useful to develop the most appropriate pathway for screening women at higher risk of breast cancer.

### Digital screening infrastructure

The current organisation of breast screening involves identifying and inviting all eligible women from GP practices to attend screening, usually in mobile screening vans or hospitals. Women not registered with a GP practice are not routinely invited.

Improvements in IT infrastructure and data interoperability [39] are needed so that all women are captured, and data recording results are readily accessible across the healthcare system before a new programme is implemented in the UK. Existing failsafe standards would need to be maintained to ensure the right women are invited to screening at the right time. There will inevitably be a trade-off between using the best possible risk prediction model and the feasibility of introducing it successfully within the current NHS BSP.

### Acceptability

A woman’s categorisation may change over time if she has repeat risk assessments. Further evaluation is needed to see whether repeat assessments and changes in screening offered are preferable to a one-off risk assessment.

The meeting considered the likelihood of a risk-stratified screening programme leading to increased anxiety and to confusion for women whose categorisation changed due to a repeated risk assessment (caused by some of the modifiable risk factors changing). Such concerns may be unfounded, even for women informed that they are at high risk [40].

Concerns were raised about the acceptability of safely extending screening intervals for women at lower risk, with one study showing 51% of women saying they would not accept less screening and 37% saying they would not accept stopping screening altogether [41].

There is some existing UK evidence supporting the need to undertake additional acceptability research [42] related to less frequent screening for low-risk women. Both women and health care professionals are less enthusiastic about reduced screening in low risk but in favour of more screening for high risk. Any change to the programme must consider safety and acceptability alongside clinical and cost effectiveness and consider how to communicate to women offered screening, for example by developing decision aids and counselling programmes [43].

There are still uncertainties about the effects that introducing risk stratification might have on screening uptake, as conducting a risk assessment may discourage some women from screening. It will be important to consider the impact of implementing AI on the accuracy, time to report, cost, and acceptability to women undergoing tailored screening.

### Screening tests

There is uncertainty on which test should be used in a stratified screening programme. Options include mammogram, automated ultrasound, handheld ultrasound, contrast enhanced mammogram, abbreviated magnetic resonance imaging and magnetic resonance imaging (MRI), but there are not yet sufficient levels of certainty on which ones would be best suited for screening in different risk groups. Supplemental ultrasound has been adopted by some national screening programmes and MRI has been found to be cost effective [44] in a large Dutch trial (DENSE trial). The UK BRAID trial [45] is comparing different supplemental modalities.

### Inequity and inequalities

Cancer incidence rates vary by ethnicity in England and the picture is complex [46]. Women from ethnic minority groups are less likely to attend breast screening compared with white British women. Estimates vary by study and by minority ethnic group [47].

Genetic differences have been suggested as a means of more accurately predicting the risk of BC. However, these polygenic risk scores are mainly based on studies of white European women and may not be as accurate for women from different ethnic backgrounds (although there are ongoing genome-wide association studies to develop multi ethnicity polygenic risk scores) [48].

It is important future discussions acknowledge this issue to ensure the accuracy of BC risk prediction for all women [49].

Another consideration for a risk-stratified NHS BSP is that women in the most deprived groups are generally less likely to participate in breast screening (relative risk (RR) 0.89 for the most deprived groups compared with the least deprived) [50] but are more likely to die from BC [1].

Although women with intellectual disabilities have the same BC incidence rate as women without intellectual disabilities, they receive fewer mammograms and have a higher mortality rate [51]. Given the barriers experienced by women with learning disabilities, reasonable adjustments and a ‘person-centred approach’ may support access to screening among this group [52].

The workshop discussed the potential for risk stratification to affect equity and/or create inequality in access. Some significant concerns were aired, although inequity and inequalities were not the focus of the session.

## Conclusion

Interactions between the UK NSC, relevant stakeholders and researchers will be crucial over the next few years to inform recommendations for breast screening in the UK, especially as the evidence base continues to shift and new findings emerge. There will be important ongoing discussions around the degree of uncertainty in model outputs and the extent to which these are deemed ‘acceptable’ in terms of informing decision-making.

The committee will continue to assess the evidence, working with researchers to highlight evidence gaps and encourage further work to develop models sufficient for informed decision-making.

Moving to risk stratification for the whole programme would be a major development. The UKNSC will need more information about the possible impact of introducing complexity into the screening programme through risk stratification compared with the simplicity of the current three-yearly screening for the vast majority.

Improved data collection is important. The news that NHSE is planning to develop a central database or register of very high-risk patients to ensure eligibility for very high risk screening is welcome. This will help ensure referrals are received by NHS BSP services [53].

If risk stratification is introduced, it should be done in phases (options include by region or by sub-group of the population) to assess real world evidence on the acceptability, safety, benefit, harms, and costs of such a change so as not to worsen inequity or create new inequalities.

Crucially, any future changes must maximise the potential to reduce BC mortality, while minimising the harms of screening and being affordable to the NHS.

## Data Availability

Not applicable.

## Abbreviations

AI: Artificial intelligence
BC: Breast cancer
BMI: Body mass index
BOADICEA: Breast and Ovarian Analysis of Disease Incidence and Carrier Estimation Algorithm
BRAID: Breast Screening Risk Adaptive Imaging for Density
GP: General practitioner
HRT: Hormone replacement therapy
IT: Information technology
MRI: Magnetic resonance imaging
NHS BSP: NHS Breast Screening Programme
NHSE: NHS England
NICE: National Institute for Health and Care Excellence
OCP: Oral contraceptive pill
QALY: Quality-adjusted life year
RCT: Randomised control trial
RR: Relative risk
UK: United Kingdom
UK NSC: UK National Screening Committee
